# Simultaneous interstitial pneumonitis and cardiomyopathy induced by venlafaxine* **

**DOI:** 10.1590/S1806-37132014000300015

**Published:** 2014

**Authors:** Pedro Gonçalo Ferreira, Susana Costa, Nuno Dias, António Jorge Ferreira, Fátima Franco

**Affiliations:** Department of Pulmonology, Coimbra Hospital and University Center, Coimbra, Portugal; Department of Cardiology, Advanced Heart Failure Treatment Unit, Coimbra Hospital and University Center, Coimbra, Portugal; Department of Anatomopathology, Coimbra Hospital and University Center, Coimbra, Portugal; Department of Pulmonology, Coimbra Hospital and University Center, Coimbra, Portugal; Department of Pulmonology, Coimbra Hospital and University Center, Coimbra, Portugal

**Keywords:** Cardiomyopathy, dilated, Lung diseases, interstitial, Antidepressive agents, second-generation, Herb-drug interactions

## Abstract

Venlafaxine is a serotonin-norepinephrine reuptake inhibitor used as an antidepressant. Interindividual variability and herb-drug interactions can lead to drug-induced toxicity. We report the case of a 35-year-old female patient diagnosed with synchronous pneumonitis and acute cardiomyopathy attributed to venlafaxine. The patient sought medical attention due to dyspnea and dry cough that started three months after initiating treatment with venlafaxine for depression. The patient was concomitantly taking *Centella asiatica* and *Fucus vesiculosus* as phytotherapeutic agents. Chest CT angiography and chest X-ray revealed parenchymal lung disease (diffuse micronodules and focal ground-glass opacities) and simultaneous dilated cardiomyopathy. Ecocardiography revealed a left ventricular ejection fraction (LVEF) of 21%. A thorough investigation was carried out, including BAL, imaging studies, autoimmune testing, right heart catheterization, and myocardial biopsy. After excluding other etiologies and applying the Naranjo Adverse Drug Reaction Probability Scale, a diagnosis of synchronous pneumonitis/cardiomyopathy associated with venlafaxine was assumed. The herbal supplements taken by the patient have a known potential to inhibit cytochrome P450 enzyme complex, which is responsible for the metabolization of venlafaxine. After venlafaxine discontinuation, there was rapid improvement, with regression of the radiological abnormalities and normalization of the LVEF. This was an important case of drug-induced cardiopulmonary toxicity. The circumstantial intake of inhibitors of the CYP2D6 isoenzyme and the presence of a CYP2D6 slow metabolism phenotype might have resulted in the toxic accumulation of venlafaxine and the subsequent clinical manifestations. Here, we also discuss why macrophage-dominant phospholipidosis was the most likely mechanism of toxicity in this case.

## Introduction

Drug-induced lung and heart disease can result from individual drug toxicity or drug-to-drug interactions, with impairment of kinetics and metabolization.^(^ ^1^ ^,^ ^2^ ^) ^The causal link between drug intake and an idiosyncratic reaction is usually difficult to recognize, especially in cases of patients treated with multiple medications.^(^ ^1^ ^,^ ^3^ ^)^ Therefore, high clinical suspicion and a thorough study are often necessary. The molecular basis of toxic lung injury is still poorly defined for the majority of the presently known offending drugs.^(^ ^2^ ^)^

We report the third case of a patient with synchronous interstitial pneumonitis and acute cardiomyopathy induced by venlafaxine, with a new insight regarding the potential lesion mechanism.

### Case report

**Figure 1:**
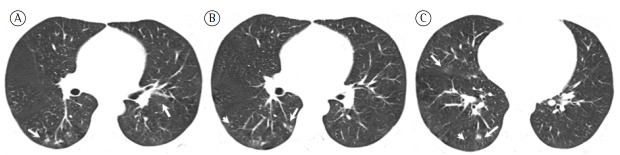
Chest CT scans at admission revealing hazy parenchymal micronodules (short arrows) with thickening of interlobular septa and subtle diffuse areas of ground glass attenuation (long arrows).

**Figure 2:**
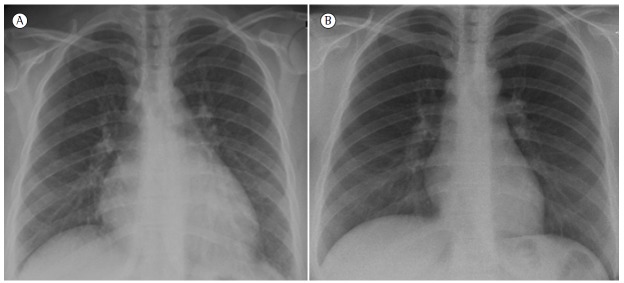
In A, a chest X-ray taken at admission showing a mixed interstitial pattern (reticular and micronodular) and cardiomegaly. In B, a chest X-ray taken at discharge showing the normalization of the lung fields and of the cardiac silhouette.

A 35-year-old female patient presented with progressive dyspnea over the previous three months, New York Heart Association (NYHA) functional class III, myalgia, and dry cough. She was hospitalized and submitted to CT angiography of the chest, which excluded pulmonary thromboembolic disease but revealed hazy parenchymal micronodules, thickening of interlobular septa, and subtle bilateral areas of ground-glass attenuation, mainly in the upper lobes, without adenopathies or pleuropericardial effusion (Figure 1). A chest X-ray revealed a mixed (reticular and micronodular) pattern and cardiomegaly (Figure 2). Simultaneously, she was diagnosed with subacute severe heart failure due to a dilated cardiomyopathy.

The patient had a history of depression and was started on a slow-release formulation of venlafaxine three months prior. For the last year, she had been taking *Centella asiatica* and *Fucus vesiculosus* as phytotherapeutic supplements for weight loss. The patient had no history of smoking, alcohol use, or illicit drug use. Previous medical examinations had been negative for heart disease, and there was neither a relevant family history nor a history of occupational exposure.

Clinical examination revealed apyrexia, mild hypotension, and an SpO_2_ of 94%. On auscultation, there were crackles at both lung bases, with a grade II/VI holosystolic murmur typical of mitral regurgitation. The jugular vein was not turgescent, nor was there hepatomegaly or peripheral edema.

Electrocardiography showed a normal sinus rhythm, ventricular premature beats, and T-wave inversion in leads V4-V6, referred to as "strain"; an initial left ventricular ejection fraction (LVEF) of 21% was identified on radionuclide angiography; biatrial dilatation, severe left ventricular enlargement (71/60 mm), and severely impaired global systolic function (LVEF = 20%) were also found; moderate right ventricular enlargement with systolic impairment-tricuspid annular plane systolic excursion of 15 mm (normal value, 15-20 mm), S' velocity of 0.06 m/s (normal value, > 0.15 m/s)-and severe functional mitral and tricuspid regurgitations were found on echocardiography.

Heart catheterization revealed normal coronary arteries and a cardiac index of 2.36 L . min^−1^ . m^−2^ (normal value, 2.6-4.2 L . min^−1^ . m^−2)^. Myocardial biopsies presented mixed cellularity without fibrosis or any other form of infiltration. Tests for DNA detection were negative for herpes simplex virus, human herpesvirus 6 (HHV-6), HHV-8, cytomegalovirus, BK virus, and Epstein-Barr virus.

Pulmonary function test results were normal. However, the diffusion capacity was slightly low (PaO_2_/FiO_2_ = 320).

Blood workup presented normal inflammatory parameters, euthyroidism, and a brain natriuretic peptide level of 963.6 pg/mL (normal value, < 100 pg/mL). Renal and hepatic parameters, complement proteins, and urinary sediment were all normal. The serological panel was negative for HIV, syphilis, *Mycoplasma* sp., *Coxiella* sp., cytomegalovirus, Epstein-Barr virus, parvovirus B19, and HHV-6. The results of testing for autoimmune disease were unremarkable.

**Figure 3:**
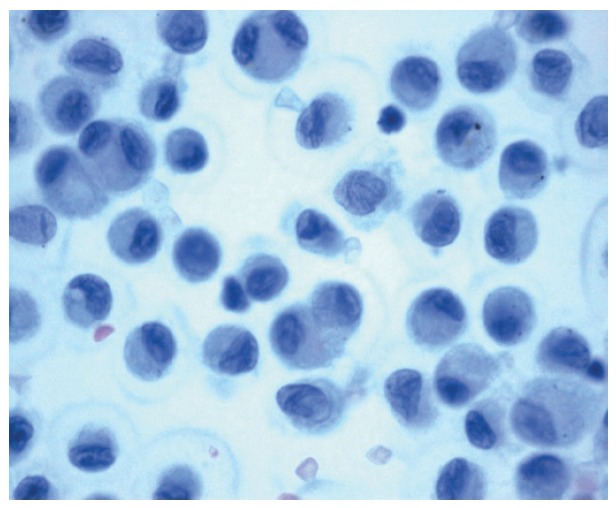
Photomicrograph showing foamy macrophages in the BAL fluid (May-Grümwald-Giemsa; magnification, ×400).

Bronchoscopy results were normal. The BAL fluid presented normal cellularity, a low CD4/CD8 lymphocyte ratio (0.7), and a massive presence of foamy macrophages (Figure 3). The microbiological study on BAL fluid was negative. No transbronchial lung biopsies were obtained, because of ventricular tachycardia during the examination.

The patient was started on standard therapy without improvement. Inotropic support was then initiated. However, no improvement was seen over the course of a week. At that time, the decision was made to discontinue venlafaxine. Two weeks later, an HRCT revealed notorious improvement, with only a few centrilobular nodules remaining in the right lower lobe. Together with that radiological upswing, there was an overall improvement, and the LVEF was up to 35% at discharge.

Four months later, the patient presented with an LVEF close to normal (50%), NYHA functional class I, maintaining only mild left ventricle enlargement (60/40 mm), and mild mitral regurgitation. Most of the cardiac medications were therefore discontinued.

## Discussion

There have been various reports on adverse effects related to venlafaxine. Acute and subacute interstitial pneumonitis have been reported^(^ ^4^ ^-^ ^7^ ^)^ as have cases of eosinophilic pneumonia,^(^ ^8^ ^,^ ^9^ ^)^ although the mechanisms involved have yet to be elucidated.

The diagnosis of drug-induced interstitial lung disease is hindered by its clinical nonspecificity, interindividual variations, the confounding effect of comorbidities, and treatment with multiple medications.^(^ ^4^ ^)^ In addition, cases of venlafaxine-induced acute heart failure have been described, some even in previously healthy patients under standard dosing regimens.^(^ ^4^ ^,^ ^10^ ^)^ One group of authors reported dilated cardiomyopathy and cardiogenic shock in a patient with previously normal heart function, with recovery of the LVEF after the discontinuation of the medication. ^(^ ^10^ ^)^ Using the Naranjo Adverse Drug Reaction Probability Scale,^(^ ^11^ ^)^ a probable link with venlafaxine was found.^(^ ^10^ ^)^ Although the mechanism of heart injury is still not fully understood, drug-induced inhibition of myocardial norepinephrine reuptake and blockade of cardiac sodium channels have been reported.^(^ ^12^ ^)^

The cytochrome P450 (CYP) superfamily is present in human lung tissue and participates in the enzymatic inactivation of numerous xenobiotics.^(^ ^13^ ^)^ Metabolic differences related to CYP polymorphisms contribute to interindividual variability reflected in drug responses and unexpected toxicity.^(^ ^2^ ^)^ One group of authors showed that, among 59 patients with drug-induced interstitial lung disease, 54 (91.5%) had at least one of the studied CYP variant genes.^(^ ^2^ ^)^ In 87% of those patients, the presence of such genes was found to be relevant to their clinical profile.^(^ ^2^ ^)^

Venlafaxine is an antidepressant that is metabolized to O-desmethylvenlafaxine (ODV) by the isoenzyme CYP2D6 and, to a lesser extent, by CYP3A4.^(^ ^14^ ^)^ For numerous psychotropic drugs, CYP2D6 is a high-affinity/low-capacity enzyme whose polymorphisms can phenotypically determine slow, extended, or rapid metabolization.^(^ ^15^ ^,^ ^16^ ^)^ Because a less functional variant might be able to precipitate severe manifestations,^(^ ^15^ ^)^ the administration of venlafaxine to CYP2D6 slow metabolizers or the coadministration with CYP2D6 inhibitor drugs carries the risk of drug accumulation and subsequent cellular/organic insult.^(^ ^16^ ^)^ It has been shown that *Centella asiatica* can induce moderate-to-strong inhibition of CYP2D6^(^ ^17^ ^)^ and that *Fucus vesiculosus* can also inhibit the cytochrome P450 enzyme complex. ^(^ ^18^ ^)^ Accordingly, although the CYP2D6 profile of our patient had not been tested, we hypothesize that she was a CYP2D6 slow metabolizer or that the inhibitory action of the concomitant use of the herbal drugs contributed to venlafaxine accumulation, leading to cardiopulmonary toxicity.

Drug-induced phospholipidosis (DIP) is characterized by intracellular accumulation of phospholipids in various body tissues and formation of lamellar bodies, leading to a foamy macrophage appearance at light microscopy. ^(^ ^19^ ^)^ It can be caused by over 50 drugs sharing a particular molecular structure, with hydrophobic and hydrophilic regions, denominated cationic amphiphilic drugs (CADs).^(^ ^20^ ^)^ The venlafaxine formulation used by our patient was analyzed by the Department of Organic Chemistry, University of Coimbra, Portugal. The patient was taking a formulation of the racemic mixture of (R/S)-1-[(2-dimethylamino)-1-(4-methoxyphenyl)ethyl]-cyclohexanol hydrochloride. Its structure combines a hydrophilic cationic component (terminal amine and tertiary alcohol groups) with a hydrophobic nonpolar component (aromatic ring methoxylation and cyclohexyl group), conferring amphiphilicity.^(^ ^21^ ^)^ In addition, it has a cationic property,^(^ ^22^ ^)^ because ODV presents an acidic phenol group (from the hydrolysis of the methyl ethers connected to the aromatic ring) and the amine group can undergo extensive intramolecular protonation, becoming a CAD by staying in zwitterion form (negative aromatic-O- and positive H+ amine groups). This fact, supported by the clinical context and the massive presence of foamy macrophages observed in the BAL fluid, strongly suggests a mechanism of venlafaxine-induced phospholipidosis.

Although DIP can occur from hours to months after the beginning of treatment,^(^ ^19^ ^)^ its precise mechanisms have yet to be completely clarified. Lysosomes act as a place of accumulation of CADs and phospholipids because of their direct connection with CADs and the inhibition of phospholipases.^(^ ^19^ ^,^ ^20^ ^)^ Because lysosomes participate in a wide variety of cellular processes, there might be ionic transport impairment, oxidative phosphorylation, heterophagy, autophagy, organelle recycling, cell membrane repairing, and cell cycle regulation. Three recognized damage patterns can occur: macrophage-dominant phospholipidosis (the commonest), parenchymal-cell-dominant phospholipidosis, and localized phospholipidosis.^(^ ^19^ ^)^

Another typical feature of DIP is its reversibility after drug discontinuation.^(^ ^1^ ^)^ Because phospholipid levels normalize and drug efflux occurs, organic, functional, and radiological abnormalities usually improve from weeks to months.^(^ ^19^ ^)^ Reversibility is usually complete, but permanent damage can subside in cases of more severe organic injury.^(^ ^2^ ^)^

The present case details the occurrence of cardiopulmonary toxicity, which was probably associated with venlafaxine. The case occurred in a previously healthy 35-year-old patient submitted to exhaustive investigation after the exclusion of other possible causes. Only two similar cases have been reported in the literature.^(^ ^4^ ^)^

We recognized the concomitant intake of two herbal drugs known to inhibit the specific metabolizing isoenzyme of venlafaxine, which would explain the accumulation of venlafaxine and ODV to toxic levels. The strong temporal connection between the drug intake and the clinical manifestations and, conversely, between the drug discontinuation and the rapid improvement in lung abnormalities and heart function, provides additional support for that hypothesis. For ethical reasons, rechallenge with venlafaxine was not carried out. After an objective analysis, considering the Naranjo Adverse Drug Reaction Probability Scale,^(^ ^11^ ^)^ we made a diagnosis of venlafaxine-induced cardiopulmonary toxicity.

A DIP could be the mechanism of toxicity, because venlafaxine is a potential CAD, as well as because there were striking quantities of foamy macrophages in the BAL fluid, a low CD4/CD8 T lymphocyte ratio in the blood and BAL fluid, and features that were consistent with a "macrophage-dominant phospholipidosis" subtype pattern.

The present case highlights the importance of high clinical suspicion for the timely recognition of drug-induced cardiopulmonary toxicity, especially in cases of initially unexplained disease. Prompt discontinuation of the drug usually results in remarkable clinical and prognostic improvement.
