# Supplementary material for: Simultaneous interstitial pneumonitis and cardiomyopathy induced by venlafaxine
Source: J Bras Pneumol. 2014 May-Jun;40(3):313–8. doi: 10.1590/S1806-37132014000300015 (PMC4109204; doi:10.1590/S1806-37132014000300015)
Supplement: Translation

# Translation: Pneumonite intersticial e miocardiopatia simultâneas induzidas por venlafaxina*

**Authors:** Pedro Gonçalo Ferreira, Susana Costa, Nuno Dias, António Jorge Ferreira, Fátima Franco

**Affiliations:** Serviço de Pneumologia, Centro Hospitalar e Universitário de Coimbra, Coimbra, Portugal; Unidade de Tratamento de Insuficiência Cardíaca Avançada, Serviço de Cardiologia, Centro Hospitalar e Universitário de Coimbra, Coimbra, Portugal; Serviço de Anatomopatologia, Centro Hospitalar e Universitário de Coimbra, Coimbra, Portugal; Serviço de Pneumologia, Centro Hospitalar e Universitário de Coimbra, Coimbra, Portugal; Serviço de Pneumologia, Centro Hospitalar e Universitário de Coimbra, Coimbra, Portugal

**Keywords:** Cardiomiopatia dilatada, Doenças pulmonares intersticiais, Antidepressivos de segunda geração/toxicidade, Toxicidade de Drogas, Interações ervas-drogas

## Abstract

A venlafaxina é um inibidor de recaptação de serotonina e noradrenalina utilizado como antidepressivo. A variabilidade individual ou interações entre fitoterápicos e fármacos podem causar toxicidade induzida por drogas. Relatamos o caso de uma paciente de 35 anos diagnosticada com pneumonite intersticial e miocardiopatia dilatada atribuídas à venlafaxina. A paciente procurou atendimento médico devido a dispneia e tosse seca, que começaram três meses após iniciar tratamento com venlafaxina para depressão. Concomitantemente tomava suplementos fitoterápicos contendo *Centella asiatica* e *Fucus vesiculosus*. A radiografia e a CT de tórax revelaram doença pulmonar parenquimatosa (micronódulos difusos e opacidades em vidro fosco) e, simultaneamente, foi diagnosticada uma miocardiopatia por ecocardiograma, que revelou uma fração de ejeção ventricular esquerda (FEVE) de 21%. Uma investigação ampla foi realizada, incluindo LBA, estudos de imagem, detecção de doenças autoimunes, cateterismo cardíaco direito e biópsia miocárdica. Após a exclusão de outras etiologias e a aplicação da Escala de Probabilidade de Reações Adversas a Medicamentos de Naranjo, foi assumido o diagnóstico de pneumonite/miocardiopatia síncronas associadas à venlafaxina. Já foi demonstrado que os suplementos fitoterápicos utilizados pela paciente podem inibir a isoenzima do complexo enzimático citocromo P450, responsável pelo metabolismo da venlafaxina. Após a descontinuação da venlafaxina, verificou-se uma rápida melhora clínica com regressão das alterações radiológicas e normalização da FEVE. Este é um importante caso de toxicidade cardiopulmonar induzida por droga. A administração circunstancial de inibidores da isoenzima CYP2D6 e a presença de um fenótipo de metabolização lenta de CYP2D6 podem ter resultado na acumulação tóxica da venlafaxina e na manifestação clínica subsequente. Aqui, é discutida a hipótese de a fosfolipidose macrofágica ser o mecanismo de toxicidade.

## Introdução

Doença pulmonar e cardíaca induzida por drogas pode resultar da toxicidade dos fármacos para o indivíduo ou da interação entre fármacos, com comprometimento da cinética e do metabolismo.^(^ ^1^ ^,^ ^2^ ^)^ O nexo de causalidade entre a administração de drogas e uma reação idiossincrática é geralmente difícil de reconhecer, especialmente em casos de pacientes tratados com múltiplos medicamentos.^(^ ^1^ ^,^ ^3^ ^)^ Portanto, são frequentemente necessários alta suspeição clínica e estudo amplo. A base molecular da lesão pulmonar tóxica ainda é pouco definida para a maioria dos medicamentos ofensivos conhecidos atualmente.^(^ ^2^ ^)^

Relatamos o terceiro caso de paciente com pneumonite intersticial e miocardiopatia dilatada induzidas pela venlafaxina, com uma nova percepção sobre o potencial mecanismo de lesão.

## Relato de caso

Paciente do sexo feminino, 35 anos de idade, apresentava dispneia progressiva nos últimos três meses, classe funcional III da *New York Heart Association* (NYHA), mialgia e tosse seca. Foi internada e submetida a angiografia por TC de tórax, que excluiu doença tromboembólica pulmonar mas revelou micronódulos parenquimatosos indistintos, espessamento dos septos interlobulares e áreas de atenuação em vidro fosco bilaterais e sutis, principalmente nos lobos superiores, sem adenopatias ou derrame pleuropericárdico (Figura 1). A radiografia de tórax revelou padrão misto (reticular e micronodular) e cardiomegalia (Figura 2). Simultaneamente, foi diagnosticada com insuficiência cardíaca subaguda grave causada por miocardiopatia dilatada.

**Figura 1:** TC de tórax na admissão hospitalar revelando micronódulos parenquimatosos indistintos (setas curtas) com espessamento dos septos interlobulares e áreas de atenuação em vidro fosco difusas e sutis (setas longas).

**Figura 2:** Em A, radiografia de tórax na admissão hospitalar mostrando padrão intersticial misto (reticular e micronodular) e cardiomegalia. Em B, radiografia de tórax na alta hospitalar mostrando a normalização dos campos pulmonares e da área cardíaca.

Apresentava história de depressão e havia iniciado tratamento com uma formulação de liberação lenta de venlafaxina há três meses. Desde o ano anterior, estava tomando suplementos fitoterápicos de *Centella asiatica* e *Fucus vesiculosus* para perda de peso. Não apresentava história de tabagismo, de uso de álcool ou de uso de drogas ilícitas. Exames médicos anteriores haviam sido negativos para doenças cardíacas, e não havia história familiar relevante nem história de exposição ocupacional.

O exame físico revelou apirexia, hipotensão leve e SpO_2_ de 94%. Na auscultação, havia estertores crepitantes bibasais, com sopro holossistólico de grau II/VI típico da regurgitação mitral. A veia jugular não apresentava inchaço; tampouco havia hepatomegalia ou edema periférico.

O eletrocardiograma mostrou ritmo sinusal normal, batimentos ventriculares prematuros e inversão da onda T em V4-V6, conhecida como "tensão"; uma fração de ejeção ventricular esquerda (FEVE) de 21% foi identificada na angiografia de radionuclídeos; também foram encontradas dilatação biatrial, dilatação grave do ventrículo esquerdo (71/60 mm) e função sistólica global gravemente comprometida (FEVE = 20%); dilatação moderada do ventrículo direito com comprometimento sistólico - excursão sistólica do plano anular da valva tricúspide de 15 mm (valor normal, 15-20 mm), velocidade de S' de 0,06 m/s (valor normal, > 0,15 m/s) - e regurgitações funcionais mitral e tricúspide graves foram encontradas no ecocardiograma.

O cateterismo cardíaco revelou artérias coronárias normais e índice cardíaco de 2,36 L . min^−1^ . m^−2^ (valor normal, 2,6-4,2 L . min^−1^ . m^−2)^. As biópsias miocárdicas apresentaram celularidade mista sem fibrose ou qualquer outra forma de infiltração. Os testes de detecção de DNA foram negativos para vírus do herpes simplex, herpesvírus humano 6 (HHV-6), HHV-8, citomegalovírus, vírus BK e vírus Epstein-Barr. 

Os resultados dos testes de função pulmonar foram normais. Porém, a capacidade de difusão mostrou-se um pouco diminuída (PaO_2_/FiO_2_ = 320).

O hemograma apresentou parâmetros inflamatórios normais, eutireoidismo e nível de peptídeo natriurético cerebral de 963,6 pg/mL (valor normal, < 100 pg/mL). Os parâmetros renais e hepáticos, as proteínas do complemento e o sedimento urinário estavam todos normais. O painel sorológico foi negativo para HIV, sífilis, *Mycoplasma* sp., *Coxiella* sp., citomegalovírus, vírus Epstein-Barr, parvovírus B19 e HHV-6. Os resultados dos testes para doenças autoimunes foram normais.

Os resultados da broncoscopia foram normais. O LBA apresentou celularidade normal, relação de linfócitos CD4/CD8 diminuída (0,7) e presença maciça de macrófagos espumosos (Figura 3). O estudo microbiológico do LBA foi negativo. Não foram obtidas biópsias pulmonares transbrônquicas, em razão de taquicardia ventricular durante o exame.

**Figura 3:** Fotomicrografia mostrando macrófagos espumosos no LBA (May-Grümwald-Giemsa; aumento, 400×).

Foi iniciado o tratamento padrão, sem melhora. Foi então iniciado suporte inotrópico. Porém, nenhuma melhora foi observada ao longo de uma semana. Na época, tomou-se a decisão de descontinuar a venlafaxina. Duas semanas depois, a TCAR revelou uma melhora notória, com a permanência de apenas alguns nódulos centrolobulares no lobo inferior direito. Juntamente com essa recuperação radiológica, verificou-se uma melhora geral, e a FEVE foi até 35% na alta hospitalar.

Quatro meses depois, a paciente apresentava FEVE próxima à normal (50%), classe funcional I da NYHA, apresentando somente dilatação leve do ventrículo esquerdo (60/40 mm) e regurgitação mitral leve. A maioria dos medicamentos cardíacos foi, portanto, descontinuada.

## Discussão

Vários trabalhos relatam efeitos adversos relacionados à venlafaxina. Há relatos de pneumonite intersticial aguda e subaguda^(^ ^4^ ^-^ ^7^ ^)^ e também de casos de pneumonia eosinofílica,^(^ ^8^ ^,^ ^9^ ^)^ embora os mecanismos envolvidos ainda não estejam esclarecidos. 

O diagnóstico da doença pulmonar intersticial induzida por drogas é dificultado por sua inespecificidade clínica, por variabilidades individuais, pelo efeito de confusão causado por comorbidades e pelo tratamento com múltiplos medicamentos.^(^ ^4^ ^)^ Além disso, foram descritos casos de insuficiência cardíaca aguda induzida pela venlafaxina, alguns até mesmo em pacientes previamente hígidos em uso de regimes padronizados de dosagem.^(^ ^4^ ^,^ ^10^ ^)^ Alguns autores relataram miocardiopatia dilatada e choque cardiogênico em um paciente com função cardíaca previamente normal, com recuperação da FEVE após a descontinuação do medicamento.^(^ ^10^ ^)^ Com o uso da Escala de Probabilidade de Reações Adversas a Medicamentos, proposta por Naranjo et al.,^(^ ^11^ ^)^ encontrou-se uma provável ligação com a venlafaxina.^(^ ^10^ ^)^ Embora o mecanismo de agressão cardíaca ainda não seja totalmente compreendido, há relatos de inibição da recaptação da noradrenalina pelo miocárdio e de bloqueio dos canais de sódio cardíacos, ambos induzidos por drogas.^(^ ^12^ ^)^

A superfamília do citocromo P450 (CYP) está presente no tecido pulmonar humano e participa da inativação enzimática de inúmeros xenobióticos.^(^ ^13^ ^)^ Diferenças metabólicas relacionadas a polimorfismos do CYP contribuem para a variabilidade refletida em respostas a drogas e toxicidade inesperada.^(^ ^2^ ^)^ Alguns autores mostraram que, entre 59 pacientes com doença pulmonar intersticial induzida por drogas, 54 (91,5%) apresentavam pelo menos um dos genes variantes do CYP estudados.^(^ ^2^ ^)^ Em 87% dos pacientes, a presença de tais genes mostrou-se relevante para o perfil clínico.^(^ ^2^ ^)^

A venlafaxina é um antidepressivo que é metabolizado em O-desmetilvenlafaxina (ODV) pela isoenzima CYP2D6 e, em menor escala, pela CYP3A4.^(^ ^14^ ^)^ Para inúmeras drogas psicotrópicas, a CYP2D6 é uma enzima de alta afinidade/baixa capacidade cujos polimorfismos podem fenotipicamente determinar uma metabolização lenta, prolongada ou rápida.^(^ ^15^ ^,^ ^16^ ^)^ Como uma variante menos funcional pode ser capaz de precipitar manifestações graves,^(^ ^15^ ^)^ a administração de venlafaxina em metabolizadores lentos de CYP2D6 ou a coadministração com inibidores de CYP2D6 traz o risco de acúmulo de fármacos e subsequente agressão celular/orgânica.^(^ ^16^ ^)^ Demonstrou-se que a *Centella asiatica* pode induzir inibição moderada a forte da CYP2D6^(^ ^17^ ^)^ e que o *Fucus vesiculosus* pode também inibir o complexo enzimático citocromo P450.^(^ ^18^ ^)^ Dessa forma, embora o perfil de CYP2D6 de nossa paciente não tenha sido testado, temos como hipótese que ela era uma metabolizadora lenta de CYP2D6 ou que a ação inibitória do uso concomitante de medicamentos fitoterápicos contribuiu para o acúmulo da venlafaxina, levando a toxicidade cardiopulmonar.

A fosfolipidose induzida por drogas é caracterizada pelo acúmulo intracelular de fosfolipídios em vários tecidos corporais e pela formação de corpos lamelares, levando a uma aparência espumosa dos macrófagos à microscopia de luz.^(^ ^19^ ^)^ Pode ser causada por mais de 50 fármacos que compartilham uma estrutura molecular específica, com regiões hidrofóbicas e hidrofílicas, denominados drogas anfifílicas catiônicas.^(^ ^20^ ^)^ A formulação de venlafaxina utilizada por nossa paciente foi analisada pelo Departamento de Química Orgânica, Universidade de Coimbra, Portugal. A paciente estava tomando uma formulação da mistura racêmica de cloridrato de (R/S)-1-[(2-dimetilamino)-1-(4-metoxifenil)etil]- ciclohexanol. Sua estrutura combina um componente catiônico hidrofílico (grupos amina terminal e álcool terciário) com um componente apolar hidrofóbico (metoxilação do anel aromático e grupo ciclohexil), conferindo anfifilicidade.^(^ ^21^ ^)^ Além disso, tem propriedade catiônica,^(^ ^22^ ^)^ pois a ODV apresenta um grupo fenol acídico (proveniente da hidrólise dos éteres metílicos ligados ao anel aromático) e o grupo amina pode sofrer grande protonação intramolecular, tornando-se uma droga anfifílica catiônica ao assumir a forma *zwitterion* (grupos amina aromática O- e amina H+). Esse fato, associado ao contexto clínico e à presença maciça de macrófagos espumosos no LBA, sugere fortemente um mecanismo de fosfolipidose induzida pela venlafaxina.

Embora a fosfolipidose induzida por drogas possa ocorrer horas ou meses após o início do tratamento,^(^ ^19^ ^)^ seus mecanismos exatos ainda não estão totalmente esclarecidos. Os lisossomos atuam como um local de acúmulo de drogas anfifílicas catiônicas e fosfolipídios por causa de sua ligação direta com as drogas anfifílicas catiônicas e da inibição das fosfolipases.^(^ ^19^ ^,^ ^20^ ^)^ Como os lisossomos participam de uma grande variedade de processos celulares, pode haver comprometimento do transporte iônico, fosforilização oxidativa, heterofagia, autofagia, reciclagem de organelas, reparação da membrana celular e regulação do ciclo celular. Três padrões reconhecidos de dano podem ocorrer: fosfolipidose macrofágica (o mais comum), fosfolipidose celular parenquimatosa e fosfolipidose localizada.^(^ ^19^ ^)^

Outra característica típica da fosfolipidose induzida por drogas é a sua reversibilidade após a descontinuação da droga.^(^ ^1^ ^)^ Como os níveis de fosfolipídios se normalizam e ocorre efluxo da droga, as alterações orgânicas, funcionais e radiológicas geralmente melhoram em semanas ou meses.^(^ ^19^ ^)^ A reversibilidade geralmente é total, mas danos permanentes podem diminuir em casos de lesão orgânica mais grave.^(^ ^2^ ^)^

O presente caso detalha a ocorrência de toxicidade cardiopulmonar, provavelmente associada à venlafaxina. O caso ocorreu em uma paciente previamente hígida de 35 anos de idade que foi submetida a investigação exaustiva após a exclusão de outras possíveis causas. Apenas dois casos semelhantes foram relatados na literatura.^(^ ^4^ ^)^

Reconhecemos a administração concomitante de dois medicamentos fitoterápicos sabidamente inibidores da isoenzima específica do metabolismo da venlafaxina, o que explicaria o acúmulo de venlafaxina e ODV a níveis tóxicos. A forte relação temporal entre a administração da droga e as manifestações clínicas e, inversamente, entre a descontinuação da droga e a rápida melhora das alterações pulmonares e da função cardíaca fornece suporte adicional a essa hipótese. Por razões éticas, não foi feita reexposição à venlafaxina. Após uma análise objetiva, considerando-se a Escala de Probabilidade de Reações Adversas a Medicamentos de Naranjo,^(^ ^11^ ^)^ foi feito o diagnóstico de toxicidade cardiopulmonar induzida pela venlafaxina. 

Uma fosfolipidose induzida por drogas poderia ser o mecanismo de toxicidade, pois a venlafaxina é uma droga anfifílica catiônica em potencial, e também porque havia grandes quantidades de macrófagos espumosos no LBA, relação de linfócitos T CD4/CD8 diminuída no sangue e no LBA e características consistentes com um padrão do subtipo "fosfolipidose macrofágica".

O presente caso destaca a importância da alta suspeição clínica para o reconhecimento oportuno da toxicidade cardiopulmonar induzida por drogas, especialmente em casos de doença inicialmente inexplicável. A descontinuação imediata da droga geralmente resulta em notável melhora clínica e do prognóstico.
